# *Lycopus lucidus Turcz* Water Extract Ameliorates the Metabolic Disorder by Up-Regulated Major Urinary Protein Expression in High-Fat Diet-Induced Obesity

**DOI:** 10.3390/cimb44050165

**Published:** 2022-05-23

**Authors:** Youngji Han, Ji-Young Choi, Eun-Young Kwon

**Affiliations:** 1 Department of Food Science and Nutrition, Kyungpook National University, 1370 San-Kyuk Dong Puk-Ku, Daegu 41566, Korea; youngji.kor.han@gmail.com; 2 Center for Food and Nutritional Genomics Research, Kyungpook National University, 1370 San-Kyuk Dong Puk-Ku, Daegu 41566, Korea; 3 Center for Beautiful Aging, Kyungpook National University, 1370 San-Kyuk Dong Puk-Ku, Daegu 41566, Korea; 4 Department of Food and Nutrition, Chosun University, 309 Pilmun-Daero, Dong-Gu, Gwangju 61452, Korea; jychoi@chosun.ac.kr

**Keywords:** diet-induced obesity, metabolic syndrome, herbal medicine, nonalcoholic fatty liver disease

## Abstract

Despite a century of research on obesity, metabolic disorders and their complications, including dyslipidemia, insulin resistance, and fatty liver disease remain a serious global health problem. *Lycopus lucidus Turcz* (LT) is a traditional medicine used for its anti-inflammatory properties that has not been evaluated for its efficacy in improving obesity. In this study, mice were fed a normal diet (*n* = 10) or obesity was induced with a high-fat diet (HFD, *n* = 20, 60% kcal from fat) for 4 weeks. The HFD mice were then divided into two groups, one of which received LT supplementation with water extract for 13 weeks [HFD (*n* = 10) or HFD with LT water extract (*n* = 10, 1.5%)]. LT reduced body and adipose tissue weight by elevating energy expenditure by increasing fatty oxidation in epididymal white adipose tissue (eWAT) and muscle. LT ameliorated dyslipidemia and hepatic steatosis by restricting lipogenesis. Additionally, LT normalized the impaired glucose homeostasis by diet-induced obesity to improve pancreatic islet dysfunction with increasing hepatic major urinary protein expression. Moreover, LT attenuated the inflammation and collagen accumulation in the liver and eWAT. In conclusion, these results suggest that LT can treat obesity-related metabolic disorders such as adiposity, dyslipidemia, hepatic steatosis, insulin resistance, and inflammation.

## 1. Introduction

The spread of coronavirus disease 2019 (COVID-19) has limited the range of human activity and has led to reduced energy consumption accompanied by increased energy intake [1]. These conditions have led to an increase in the obese population. Obesity is well known to be a major risk factor for common diseases such as type 2 diabetes, cardiovascular disease, dyslipidemias, and cancers [2]. In addition, obesity decreases lung capacity and commonly exacerbates the severity of respiratory diseases, including COVID-19 [3,4]. Moreover, overweight and obese people are a vulnerable group, at greater risk during the COVID-19 pandemic [5]. A COVID-19 case study suggested that risk of hospitalization, intensive care unit admission, invasive mechanical ventilation, and death are higher with increasing BMI [6]. In the special situation of COVID-19, it is difficult to increase energy consumption for body weight loss. Perhaps the best way to lose weight is to eat functional foods.

Although many healthy, functional foods have been developed and researched to improve obesity, there is high demand for new food materials to reduce body fat. The leaves of *Lycopus lucidus Turcz* (LT), a member of the *Lamiaceae* family, have been used for centuries as a traditional phytomedicine and dietary herb with anti-inflammatory, cardiac, sedative, wound-healing, pain relief, and tonic effects [7,8]. LT has anti-oxidative and anti-inflammatory effects and could inhibit high glucose-induced vascular inflammation [9,10].

In the present study, we explored the potential of this herb as a dietary or supplemental source for a modulator of obesity-associated metabolic disorders. We investigated the molecular mechanism of the anti-metabolic syndrome action of the LT, *Lamiaceae*, and herbaceous perennial plant extract in diet-induced obese mice. This study provides a potential application for LT as a modulator of obesity-associated metabolic disorders in functional foods.

## 2. Materials and Methods

### 2.1. Preparation of Substance

LT was provided from PUREMIND Co., Ltd., Yeongcheon, Korea. The raw material, LT, was poured into purified water, heated, and pressure-extracted two times under 105 °C for 5 h. The extracts were first filtered through a screen (60 mesh), and the remaining residue was completely removed through a second fine filter (CP 0.1 μm). The extracted liquid was concentrated and freeze-dried, yielding a 15.44% extract. Table 1 shows the result of component analysis using LT extract. Total polyphenolic content was expressed as mg gallic acid equivalents per 100 g dry weight (mg GAE/100 g). Total flavonoid content was expressed as mg quercetin equivalents per 100 g dry weight.

### 2.2. Experimental Animals and Diets

Male C57BL/6J mice (four weeks old, *n* = 35) were obtained from the Jackson Laboratory (Bar Harbor, ME, USA). All mice were individually housed under a constant temperature (22 ± 2 °C) with a 12 h light/dark cycle and fed a normal chow diet (SAFE40 + RMM, Scientific animal food & engineering, Amersfoort, The Netherlands) for 1 week after arrival. They were then randomly divided into a control group (ND, fed a normal diet, *n* = 10) and a high-fat diet group (HFD with 60% of kilocalories from fat, *n* = 20) for 4 weeks. After 4 weeks of obesity induction, the HFD-fed mice were again randomly divided into two groups and fed either a HFD (*n* = 10) or HFD with 1.5% (*w*/*w*) LT water extract (LT, *n* = 10) for 13 weeks (Supplementary Table S1). The mice had free access to food and water during the experimental period, and their body weight and food intake were measured weekly. At the end of the experiment, all mice were anesthetized with isoflurane and sacrificed after a 12 h fast. Blood was collected from the inferior vena cava for plasma biomarker measurement. The liver and adipose tissue were removed, rinsed with physiological saline, immediately frozen in liquid nitrogen, and stored at −80 °C until use. The animal protocols were approved by the Ethics Committee of Kyungpook National University (Approval No. 2017-0117).

### 2.3. Energy Expenditure

Energy expenditure was measured using an indirect calorimeter (Oxylet; Panlab, Cornella, Spain) in three randomly selected mice per group. The mice were placed into individual metabolic chambers at 25 °C, with free access to food and water. O_2_ and CO_2_ analyzers were calibrated with high-purity gas. Whole-body oxygen consumption (VO_2_) and carbon dioxide production (VCO_2_) were recorded at 3 min intervals using a computer-assisted data acquisition program (AD Instrument, Sydney, Australia) over a 24 h period, and the data were averaged for each mouse. Energy expenditure (EE) was calculated according to the following formula:

EE (kcal∙day^−1^∙bodyweight^−0.75^) = VO_2_ × 1.44 × [3.815 + (1.232 × VCO_2_/VO_2_)] (1)

### 2.4. Histology and Immunohistochemistry

Liver and epididymal white adipose tissue were removed and fixed in 10% formalin. All fixed tissues were processed routinely for paraffin embedding; 4-μm sections were prepared and stained with hematoxylin and eosin (H&E) and Masson’s trichrome stain. For the immunohistochemistry of pancreatic β-cells, the islet was sectioned and fixed in 10% formalin. For immunohistochemistry, the islet was sectioned, fixed in hydrogen peroxide, and washed in citrate buffer (pH 6.0). These sections were treated with a blocking reagent (Ultra-Tech HRP, Jacksonville, FL, USA) to prevent nonspecific binding and incubated with monoclonal antibodies against insulin and glucagon (Santa Cruz Biotech, Santa Cruz, CA, USA). Antibody reactivity was detected using HRP-conjugated biotin-streptavidin complexes and developed with diaminobenzidine tetrahydrochloride substrate. All stained areas were viewed using an optical microscope (Nikon, Tokyo, Japan) with a magnifying power of ×200.

### 2.5. Plasma Biomarkers

The plasma total cholesterol (TC), high density lipoprotein cholesterol (HDL-C), and triglyceride (TG) concentrations were determined using enzymatic kits (Asan Pharm Co., Seoul, Korea). The apolipoprotein A-I (Apo A-I) and apolipoprotein B (Apo B) levels were also measured using enzymatic kits (Eiken, Tokyo, Japan). Plasma levels of free fatty acid (FFA) and phospholipid were measured using the Wako enzymatic kit (Wako Chemicals, Richmond, VA, USA). The non-HDL-C was calculated as follows: (Total-C) − (HDL-C). The glutamic oxaloacetic transaminase (GOT) and glutamic pyruvic transaminase (GPT) levels were measured using a commercial kit (Asan Pharm Co., Seoul, Korea). Plasma hormones (insulin and glucagon) were measured with a MILLIPLEX detection kit (Merck Millipore, Billerica, MA, USA). All samples were assayed in duplicate and analyzed with a Luminex 200 Labmap system (Luminex, Austin, TX, USA).

### 2.6. Fasting Blood Glucose, Intraperitoneal Glucose Tolerance Test, and Homeostatic Index of Insulin Resistance

After 12 h fasting, blood glucose concentration was measured every 4 weeks from the tail vein using a glucose analyzer, One Touch Select Plus (Lifescan Inc., Malvern, PA, USA). The intraperitoneal glucose tolerance test (IPGTT) was performed at the 16th week. After a 12 h fast, the mice were intraperitoneally injected with glucose (0.5 g/kg body weight), and blood glucose was measured at the tail vein at 0, 30, 60, and 120 min after the injection. The homeostatic index of insulin resistance (HOMA-IR) was calculated as follows: HOMA-IR = [fasting glucose (mmol/L) × fasting insulin (μL U/mL)]/22.5.

### 2.7. Hepatic Lipid Content

Hepatic lipids were extracted [11], and the dried lipid residues were dissolved in 1 mL ethanol for triglyceride and cholesterol assays. Triton X-100 and a sodium cholate solution in distilled water were added to 200 μL of the dissolved lipid solution for emulsification. The triglyceride, cholesterol, and fatty acid concentrations were conducted using the same enzymatic kit for plasma analyses.

### 2.8. Hepatic Lipid-Regulating and Glucose-Regulating Enzyme Activities

Enzymes were prepared according to Hulcher and Oleson with a slight modification [12]. Protein concentrations were determined by the Bradford method using bovine serum albumin as the standard [13]. Phosphatidate phosphohydrolase (PAP) activity was measured using the technique of Walton & Possmayer [14]. Malic enzyme (ME) activity was measured according to Ochoa [15]. Phosphoenolpyruvate carboxykinase (PEPCK) activity was monitored for oxaloacetate synthesis using the spectrophotometric assay developed by Bentle and Lardy [16].

### 2.9. Real-Time qPCR

The liver was collected from three mice randomly selected from each of the normal diet (ND), HFD, and LT groups. Total RNA was extracted using TRIZOL reagent (Invitrogen Life Technologies, Grand Island, NY, USA) and reverse transcribed using the PrimeScript RT reagent kit with gDNA Eraser (TAKARA, Maebashi, Japan). Transcript expression was quantified by real-time quantitative PCR (RT-qPCR), using the SYBR green PCR kit (Qiagen, Hilden, Germany) and the CFX96TM real-time system (Bio-Rad, Hercules, CA, USA). Gene-specific mouse primers are listed in Supplementary Table S2. Cycle threshold (Ct) data were normalized using glyceraldehyde 3-phosphate dehydrogenase (GAPDH), and relative gene expression was calculated by the 2^−ΔΔCt^ method [17].

### 2.10. Hepatic mRNA Sequencing

HiSeq Illumina sequencing was performed by (Macrogen, Seoul, Korea). The TruSeq Stranded mRNA Sample Preparation Protocol provided by Illumina was followed [18]. The HiSeq4000 System Protocol was provided by Illumina (https://support.illumina.com/content/dam/illumina-support/documents/documentation/system_documentation/hiseq4000/hiseq-4000-system-guide-15066496-06.pdf (accessed on 28 April 2022)). All samples were indexed so that pools can be run across 15 lanes of a four-laned Illumina flow cell, providing an estimated 20–30 million single-end reads per sample.

### 2.11. Statistical Analysis

Data are presented as the mean ± standard error of the mean (SE). All statistical analyses were performed using SPSS version 23.0 (SPSS, Chicago, IL, USA). Significant differences between the ND and HFD groups were determined using Student’s *t*-test, and significant differences between the HFD and LT groups were also determined using Student’s *t*-test. Differences were considered statistically significant at *p* < 0.05.

## 3. Results

### 3.1. LT Supplementation Reduced Body Weight and Body Fat Mass

**Figure 1:**
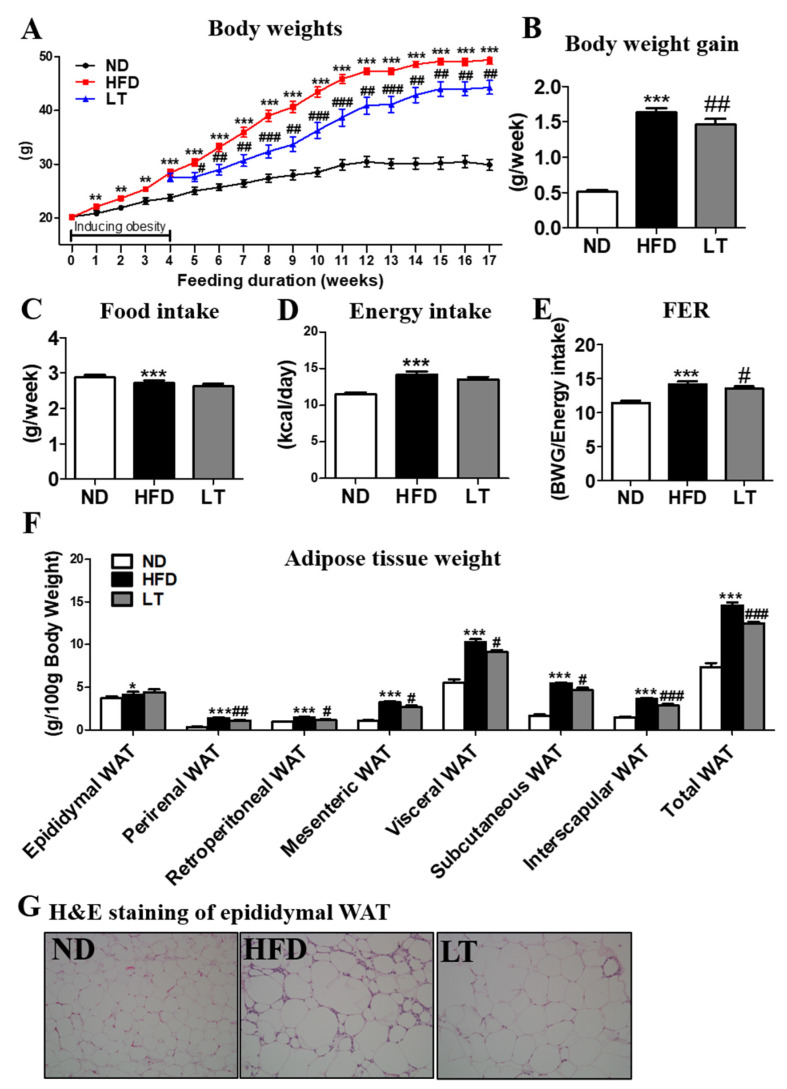
Effect of LT water extract supplement in diet-induced obesity on body weight (**A**), body weight gain (**B**), food intake (**C**), energy intake (**D**), food efficiency ratio (**E**), adipose tissue weight (**F**), adipose tissue morphology (200× magnification) (**G**); Data are the mean ± SE. Significant differences between HFD versus ND are indicated: * *p* < 0.05, ** *p* < 0.01, *** *p* < 0.001. Significant differences between HFD and LT are indicated: # *p* < 0.05, ## *p* < 0.01, ### *p* < 0.001. ND, normal diet (AIN-93G); HFD, high-fat diet (60% kcal from fat); LT, HFD + *Lycopus lucidus Turcz* water extract (1.5%, *w*/*w*); FER, food efficiency ratio: body weight gain/energy intake per day; WAT, white adipose tissue.

To induce obesity, mice were fed HFD 4 weeks before LT supplementation. At the 4th week, body weight was significantly higher in the HFD group than the ND group with *p* < 0.001 (Figure 1A). LT supplementation was initiated in the 4th week. From the 5th week to follow-up, we observed a significant reduction in body weight and weight gain in the supplement group compared with the HFD group that did not receive the supplement (Figure 1A,B). There were no significant differences in food and energy intake between the HFD and LT groups; however, the food efficiency ratio was significantly lowered in the LT group compared to that in the HFD group (Figure 1C–E). The HFD group showed a dramatic increase in all types of adipose tissue weight, whereas LT supplementation significantly decreased all adipose tissue weights except for the epididymal fat-pad mass (Table 2).

Plasma lipid profiles are shown in Table 1. Plasma levels of TG, phospholipid, total cholesterol, HDL cholesterol, non-HDL cholesterol, ApoA-1, and ApoB were significantly higher in the HFD group than the ND group; however, LT supplementation significantly suppressed these values compared with the HFD group.

Similarly, morphological observations of lipid formation in the epididymal white adipose tissue (WAT) showed that the HFD group had larger areas of lipid formation than the ND group (Figure 1G). However, LT supplementation reduced adipocyte size in epididymal WAT.

### 3.2. LT Supplementation Increased EE and Regulated mRNA Expression of WAT and Muscle

**Figure 2:**
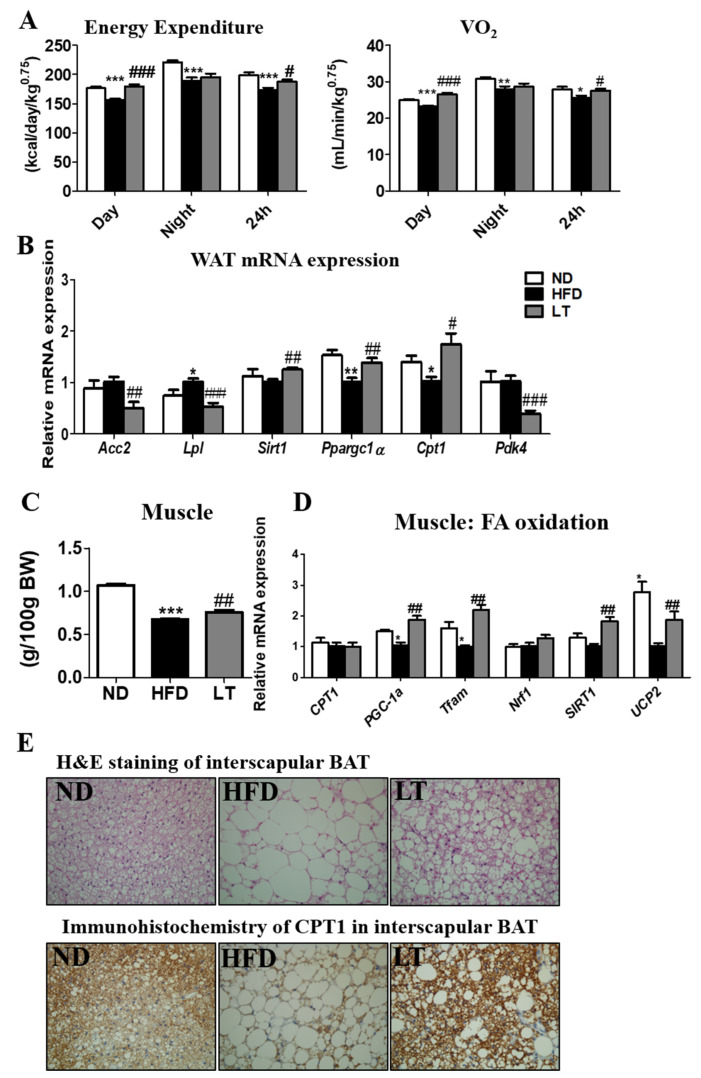
Effect of LT water extract supplement in diet-induced obesity on energy expenditure and VO_2_ (**A**), WAT mRNA expression (**B**), muscle weight (**C**), muscle mRNA expression (**D**), epididymal white adipose tissue and interscapular brown adipose tissue morphology (200× magnification) (**E**); Data are the mean ± SE. Significant differences between HFD versus ND are indicated: * *p* < 0.05, ** *p* < 0.01, *** *p* < 0.001. Significant differences between HFD versus LT are indicated: # *p* < 0.05, ## *p* < 0.01, ### *p* < 0.001. ND, normal diet (AIN-93G); HFD, high-fat diet (60% kcal from fat); LT, HFD + *Lycopus lucidus Turcz* water extract (1.5%, *w*/*w*); VO_2_, O_2_ consumption; Acc2, acetyl-CoA carboxylases; Lpl, lipoprotein lipase; Sirt1, Sirtuin1; Ppargc1a; peroxisome proliferator-activated receptor gamma coactivator 1-alpha; Cpt1, carnitine palmitoyl transferase1; Pdk4, pyruvate dehydrogenase kinase 4; ppara, peroxisome proliferator-activated receptor alpha; CD36, cluster of differentiation 36; Slc2a4, solute carrier family 2 member 4; PGC-1a, peroxisome proliferator-activated receptor gamma coactivator-1 alpha; tfam, transcription factor A, Nrf1, nuclear respiratory factor 1; UCP2; uncoupling protein 2.

As shown in Figure 2A, HFD-fed mice showed significantly decreased EE with high O_2_ consumption, whereas LT supplementation significantly increased the EE and VO_2_ compared to the HFD group. In epididymal eWAT, LT supplementation significantly decreased the expression of Acc2, Lpl, and Pdk4 and increased the expression of Sirt1, Ppargc1a, and carnitine palmitoyl transferase1 (Cpt1), compared with the HFD group (Figure 2B).

Likewise, LT significantly suppressed muscle loss associated with the HFD (Figure 2C). Also, muscle mRNA expression related fatty acid oxidation, CPT1, PGC-1a, Tfam, Nrf1, SIRT1, and UCP2, were significantly increased by LT supplementation (Figure 2D).

H&E staining showed an increase in fats in interscapular brown adipose tissue (BAT) in the HFD group compared with that in the ND group; however, LT supplement decreased (Figure 2E). Furthermore, immunohistochemistry analysis showed that LT increased CPT1 expression in interscapular BAT.

### 3.3. LT Supplementation Improved Hepatic Steatosis

**Figure 3:**
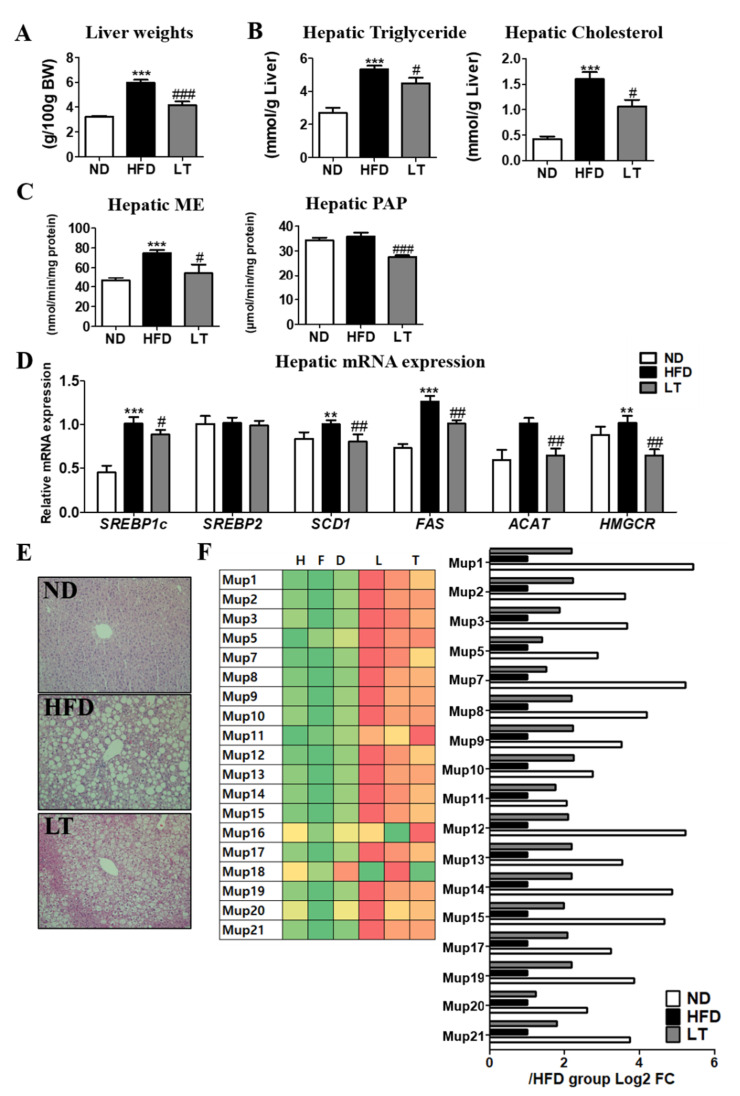
Effect of LT water extract supplement in diet-induced obesity on liver weight (**A**), hepatic lipid contents (**B**), hepatic lipid-regulating enzyme activities (**C**), hepatic mRNA expression (**D**), hepatic morphology (200× magnification) (**E**) hepatic mRNA expression of MUPs using mRNA-sequencing (**F**); Data are the mean ± SE. Significant differences between HFD versus ND are indicated: ** *p* < 0.01, *** *p* < 0.001. Significant differences between HFD versus LT are indicated: # *p* < 0.05, ## *p* < 0.01, ### *p* < 0.001. ND, normal diet (AIN-93G); HFD, high-fat diet (60% kcal from fat); LT, HFD + *Lycopus lucidus Turcz* water extract (1.5%, *w*/*w*); ME, malic enzyme; PAP, phosphatidic acid phosphatase; SREBP, sterol regulatory element binding proteins; SCD1, Stearoyl CoA Desaturase 1; FAS, fatty acid synthetase; ACAT, Acyl-CoA cholesterol acyltransferase; HMGCR, 3-hydroxy-3-methyl-glutaryl-coenzyme A reductase.

LT supplementation significantly reduced liver weight and lowered hepatic triglyceride and cholesterol levels (Figure 3A,B). Additionally, hepatic ME and phosphatidic acid phosphatase activities were significantly increased compared with the HFD group (Figure 3C). Moreover, hepatic mRNA expression, sterol regulatory element binding proteins (SREBP)1c, SREBP2, FAS, Acyl-CoA cholesterol acyltransferase (ACAT), and 3-hydroxy-3-methyl-glutaryl-coenzyme A reductase (HMGCR) were significantly decreased by LT supplementation compared with the HFD group (Figure 3D). Consistent with these results, H&E staining revealed that LT suppressed hepatic lipid accumulation compared with HFD-fed mice (Figure 3E). In mRNA sequencing analysis using hepatic tissue, mRNA expression-related major urinary protein (MUP) synthesis decreased by HFD-induced obesity; however, LT supplement decreased that of the HFD group (Figure 3F).

### 3.4. LT Supplement Improved the Impaired Glucose Metabolism-Related Obesity

**Figure 4:**
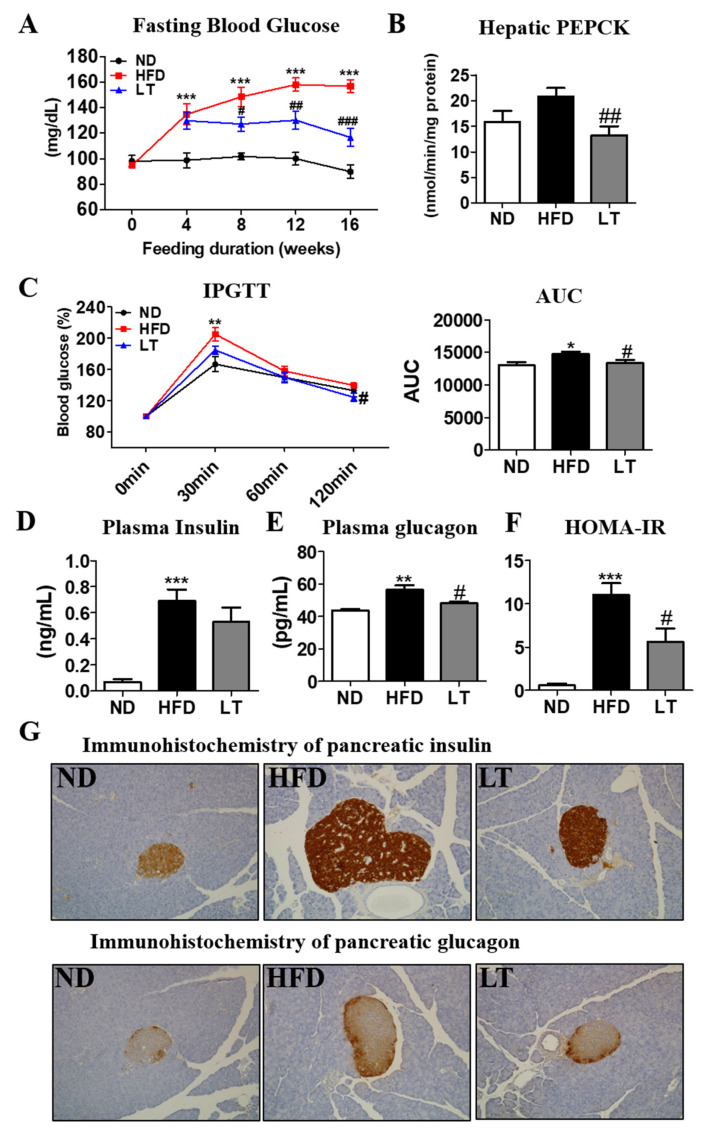
Effect of LT water extract supplement in diet-induced obesity on energy expenditure and fasting blood glucose (**A**), hepatic PEPCK (**B**), IPGTT and AUC (**C**), plasma insulin (**D**), plasma glucagon (**E**), HOMA-IR (**F**), pancreatic immunohistochemistry analysis (200× magnification) (**G**). Data are the mean ± SE. Significant differences between HFD versus ND are indicated: * *p* < 0.05, ** *p* < 0.01, *** *p* < 0.001. Significant differences between HFD versus LT are indicated: # *p* < 0.05, ## *p* < 0.01, ### *p* < 0.001. ND, normal diet (AIN-93G); HFD, high-fat diet (60% kcal from fat); LT, HFD + *Lycopus lucidus Turcz* water extract (1.5%, *w*/*w*); PEPCK, phosphoenolpyruvate carboxykinase; IPGTT, intraperitoneal glucose tolerance test; AUC, area under the curve; HOMA-IR, homeostasis model assessment of insulin resistance; PEPCK, phosphoenolpyruvate carboxykinase.

Fasting blood glucose (FBG) in LT group mice was significantly lower in HFD-fed mice beginning in the 4th week of feeding with decreased hepatic PEPCK activity (Figure 4A,B). IPGTT and area under the curve (AUC) results indicated LT-ameliorated glucose intolerance (Figure 4C). There was no significant difference in plasma insulin levels between the HFD and LT group; however, plasma glucagon and HOMA-IR were significantly decreased in the LT group (Figure 4D–F). Immunohistochemical staining of the pancreatic tissue for insulin and glucagon revealed that pancreatic islet hypertrophy caused by HFD was prevented by LT treatment (Figure 4G).

### 3.5. LT Supplementation Ameliorated Inflammation

**Figure 5:**
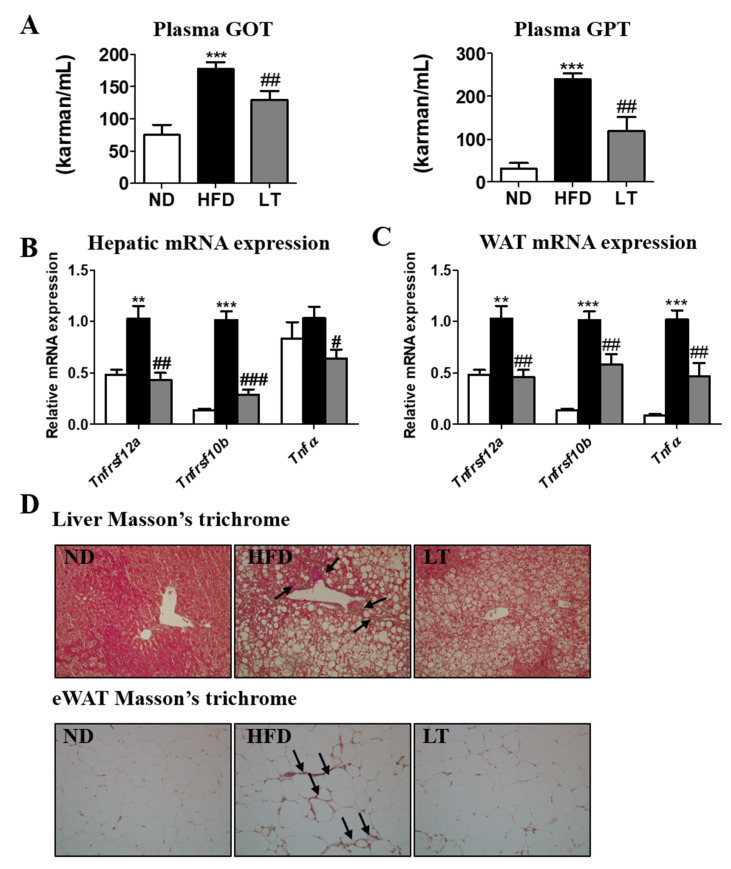
Effect of LT water extract supplement in diet-induced obesity on plasma GOT and GPT (**A**), hepatic mRNA expression (**B**), epididymal adipose tissue mRNA expression (**C**), and Masson’s trichrome stained transverse-section of liver and epididymal fat (200× magnification) (**D**) in C57BL/6J mice fed a high-fat diet; Data are the mean ± SE. Significant differences between HFD versus ND are indicated: ** *p* < 0.01, *** *p* < 0.001. Significant differences between HFD versus LT are indicated: # *p* < 0.05, ## *p* < 0.01, ### *p* < 0.001. ND, normal diet (AIN-93G); HFD, high-fat diet (60% kcal from fat); LT, HFD + *Lycopus lucidus Turcz* water extract (1.5%, *w*/*w*). GOT, glutamic oxaloacetic transaminase; GPT, glutamic pyruvic transaminase; tnfrsf, tumor necrosis factor receptor superfamily member; tnfα, tumor necrosis factor-α.

Hepatotoxicity was indicated by plasma levels of GOT and GPT; HFD significantly increased those values, whereas LT supplementation significantly reduced them (Figure 5A). In addition, hepatic and eWAT mRNA expression-related Tnfa were significantly increased in HFD-fed mice and significantly decreased in LT-treated mice (Figure 5B,C).

As shown in Figure 5D, we performed Masson’s trichrome (MT) staining to investigate fibrosis and collagen accumulation in the liver and eWAT. MT staining showed a greater accumulation of collagen in the liver of the HFD group than in the ND group. On the other hand, LT supplementation reduced collagen accumulation compared to the HFD group and was similar to that of the ND group.

## 4. Discussion

This study evaluated the effect of LT water extract on metabolic disorders caused by obesity and its complications. In this study, LT supplementation ameliorated the adiposity and hyperlipidemia caused by diet-induced obesity without changes in food and energy intake. Increasing metabolic energy consumption is the primary solution to the obesity problem [19]. Muscle, the representative energy-consuming organ, is unique in its ability to rapidly increase its rate of metabolic energy consumption in situations where explosive contractions are required [20,21]. Lipid oxidation of fatty acids is the main energy resource in muscle energy metabolism [22]; however, it is reduced in obese statuses [23]. In this study, LT supplementation increased muscle expression of transcripts related to fatty acid oxidation (CPT1, PGC-1a, Tfam, Nrf1, SIRT1, and UCP2) without obesity-induced muscle loss. BAT and thermogenic adipocytes release the energy in the form of heat through the high expression of UCP1. LT supplement increased the UCP expression in BAT effectively. Consistent with these results, body weight and body fat were significantly decreased with elevated metabolic rates in LT-supplemented mice.

There is much evidence that obesity can induce liver dysfunction and hepatic steatosis [24]. Excessive energy intake through a HFD is stored in the form of lipid droplets in the liver and adipose tissue to maintain metabolic energy homeostasis [25,26]. Lee et al. reported that LT ethanol extract attenuated hepatic steatosis in vitro and in vivo [27]. Consistent with these results, despite the different extraction conditions, this study showed an increase in liver weight due to excessive fat accumulation in the HFD group. However, LT supplementation suppressed hepatic lipid accumulation and mRNA expression associated with lipid metabolism and decreased the hepatotoxicity index GOT and GPT. LT supplementation increased surplus energy consumption due to a HFD, suggesting a reduced intake of surplus energy into the liver.

Furthermore, improved hepatic steatosis by LT supplementation was strongly associated with improved insulin resistance. The liver is a major organ that regulates glucose homeostasis, but hepatic steatosis caused by diet-induced obesity interferes with the regulation of glucose metabolism [28,29]. In our study, FBG and AUC in IPGTT were dramatically ameliorated by LT supplement with hepatic steatosis improvement. Moreover, an increase in excessive gluconeogenesis is also reported to contribute to the development of insulin resistance. In our study, LT supplementation decreased hepatic PEPCK activity and improved pancreatic islet hypertrophy. Our findings suggested that LT might have a protective role in insulin resistance by normalizing glucose metabolism in diet-induced obesity.

MUPs are produced and secreted from the liver and then excreted in the urine and serve as a metabolic signal regulating glucose and lipid metabolism [30]. Circulating MUPs ameliorates impaired glucose homeostasis and promotes the energy expenditure by improving mitochondrial dysfunction [31]. In genetic obesity, ob/ob and db/db, serum levels of MUPs and its mRNA expression were significantly decreased [31,32]. In our study, hepatic mRNA expression of MUPs were significantly decreased in diet-induced obesity also; however, LT supplement dramatically increased them. Consistent with this result, LT supplement significantly increased the energy expenditure with up-regulating of mRNA expression-related fatty acid oxidation. Moreover, LT supplement LT alleviates insulin resistance and glucose intolerance. Taken together, LT supplement improved the metabolic disorder by diet-induced obesity by up-regulating mRNA expression of MUPs.

Many studies have reported that LT has anti-inflammatory properties for various in vitro models [9,10,27]. LT water extract decreases high glucose-induced vascular inflammation by suppressing reactive oxygen species and NF-ĸB activation in human umbilical vein endothelial cells [10]. Additionally, LT ethanol extract decreased TNF-α expression in a HFD diet-induced obese model [27]. Likewise, our findings indicated that LT water extract has anti-inflammatory properties effected by reducing TNF-a mRNA expression and attenuating fibrosis in the liver and eWAT. Moreover, LT contains several antioxidant polyphenols and flavonoids with great free radical-scavenging ability. Luteolin, the major component of LT extract, has great antioxidant and anti-inflammatory properties as it inhibits TNF-α expression. Therefore, dietary LT supplementation attenuates inflammation caused by diet-induced obesity and inhibits liver and adipose tissue fibrosis.

Our finding suggested that a 1.5% LT (*w*/*w*) supplement ameliorated the diet-induced obesity. When translating results from pharmacological challenges in mice models to humans, one must take into account the differences between mice and human metabolism. The doses used in our study would reflect 18.24 mg/kg*body weight for humans according to the body surface area normalization method [10]. In other words, the effective dose of LT supplement for human with a mean body weight of 60 kg presented as 1.09 g per day.

## 5. Conclusions

Our findings indicate the effects of LT supplementation on metabolic dysregulation in diet-induced obese mice. LT improves adiposity by increasing energy consumption and lipid oxidation and suppresses fatty liver by reducing lipid uptake into the liver. Furthermore, an LT supplement contributes to the recovery of impaired glucose homeostasis and alleviates inflammation and fibrosis with up regulating mRNA expression of MUPs caused by diet-induced obesity.

## Figures and Tables

**Table 1:** Total phenolic, flavonoid, and luteolin content in the *Lycopus lucidus Turcz* water extract.

	Amount
Total polyphenolic content	480.86 ± 1.70 mg (GAE)/g
Total flavonoid content	76.61 ± 0.16 mg (QE)/g
Luteolin	2.426 mg/100 g

Values represent mean ± SE.

**Table 2:** Effect of *Lycopus lucidus Turcz* water extract supplement for 13 weeks on plasma lipid profiles in C57BL/6J mice fed a HFD.

	ND	HFD	LT
FFA (mmol/L)	0.44 ± 0.05	0.40 ± 0.02	0.36 ± 0.02
TG (mmol/L)	1.15 ± 0.10	1.61 ± 0.11 **	1.50 ± 0.11
PL (mg/dL)	228.39 ± 10.82	352.49 ± 11.46 ***	253.27 ± 10.11 ^###^
Total-C (mmol/L)	3.59 ± 0.09	7.85 ± 0.26 ***	4.85 ± 0.10 ^###^
HDL-C (mmol/L)	0.59 ± 0.05	1.39 ± 0.07 ***	0.99 ± 0.09 ^##^
nonHDL-C (mmol/L)	3.13 ± 0.20	6.49 ± 0.26 ***	4.01 ± 0.23 ^###^
Apo B (mg/dL)	6.39 ± 0.37	8.51 ± 0.51 **	6.42 ± 0.31 ^##^
Apo A-1(mg/dL)	63.72 ± 1.85	68.64 ± 0.99 *	62.10 ± 1.46 ^##^
Apo B/Apo A-1	0.102 ± 0.007	0.127 ± 0.006 *	0.107 ± 0.004 ^#^

Data are the mean ± SE. Significant differences between HFD versus ND are indicated: * *p* < 0.05, ** *p* < 0.01, *** *p* < 0.001. Significant differences between HFD versus LT are indicated: ^#^ *p* < 0.05, ^##^ *p* < 0.01, ^###^ *p* < 0.001. ND, normal diet (AIN-93G); HFD, high-fat diet (60% kcal from fat); LT, HFD + *Lycopus lucidus Turcz* water extract (1.5%, *w*/*w*); FFA, free fatty acid; TG, triglyceride; Total-C, total-cholesterol; HDL-C, high density lipoprotein cholesterol; nonHDL-C: (Total-C) − (HDL-C); ApoA-1, apolipoprotein A-1; Apo B, apolipoprotein B; HTR, (HDL-C/Total-C) × 100; AI, Atherogenic index, [(total-C) − (HDL-C)]/HDL-C.

## Data Availability

Not applicable.

## Supplementary Materials

The following supporting information can be downloaded at: https://www.mdpi.com/article/10.3390/cimb44050165/s1, Table S1: Experimental Diets; Table S2: Primer sequences used for RT-qPCR.

## References

[1] Polero P., Rebollo-Seco C., Adsuar J.C., Pérez-Gómez J., Rojo-Ramos J., Manzano-Redondo F., Garcia-Gordillo M., Carlos-Vivas J. (2021). Physical activity recommendations during COVID-19: Narrative review. Int. J. Environ. Res. Public Health.

[2] Gesta S., Tseng Y.-H., Kahn C.R. (2007). Developmental origin of fat: Tracking obesity to its source. Cell.

[3] Gao F., Zheng K.I., Wang X.-B., Sun Q.-F., Pan K.-H., Wang T.-Y., Chen Y.-P., Targher G., Byrne C.D., George J. (2020). Obesity is a risk factor for greater COVID-19 severity. Diabetes Care.

[4] Simonnet A., Chetboun M., Poissy J., Raverdy V., Noulette J., Duhamel A., Labreuche J., Mathieu D., Pattou F., Jourdain M. (2020). High prevalence of obesity in severe acute respiratory syndrome coronavirus-2 (SARS-CoV-2) requiring invasive mechanical ventilation. Obesity.

[5] Cuschieri S., Grech S. (2020). Obesity population at risk of COVID-19 complications. Glob. Health Epidemiol. Genom..

[6] Kompaniyets L., Goodman A.B., Belay B., Freedman D.S., Sucosky M.S., Lange S.J., Gundlapalli A.V., Boehmer T.K., Blanck H.M. (2021). Body mass index and risk for COVID-19—Related hospitalization, intensive care unit admission, invasive mechanical ventilation, and death—United States, March–December 2020. Morb. Mortal. Wkly. Rep..

[7] Yang X., Zhao Y., He N., Croft K.D. (2010). Isolation, characterization, and immunological effects of α-galacto-oligosaccharides from a new source, the herb *Lycopus lucidus Turcz*. J. Agric. Food Chem..

[8] Lu Y.-h., Huang J.-h., Li Y.-c., Ma T.T., Sang P., Wang W.J., Gao C.Y. (2015). Variation in nutritional compositions, antioxidant activity and microstructure of *Lycopus lucidus* Turcz. root at different harvest times. Food Chem..

[9] Ślusarczyk S., Hajnos M., Skalicka-Woźniak K., Matkowski A. (2009). Antioxidant activity of polyphenols from *Lycopus lucidus* Turcz. Food Chem..

[10] Lee Y.J., Kang D.G., Kim J.S., Lee H.S. (2008). *Lycopus lucidus* inhibits high glucose-induced vascular inflammation in human umbilical vein endothelial cells. Vasc. Pharmacol..

[11] Folch J., Lees M., Sloane-Stanley G. (1957). A simple method for the isolation and purification of total lipids from animal tissues. J. Biol. Chem..

[12] Hulcher F.H., Oleson W.H. (1973). Simplified spectrophotometric assay for microsomal 3-hydroxy-3-methylglutaryl CoA reductase by measurement of coenzyme A. J. Lipid Res..

[13] Bradford M.M. (1976). A rapid and sensitive method for the quantitation of microgram quantities of protein utilizing the principle of protein-dye binding. Anal. Biochem..

[14] Walton P.A., Possmayer F. (1984). The role of Mg^2+^-dependent phosphatidate phosphohydrolase in pulmonary glycerolipid biosynthesis. Biochim. Biophys. Acta (BBA) Lipids Lipid Metab..

[15] Ochoa S., Mehler A.H., Kornberg A. (1948). Biosynthesis of dicarboxylic acids by carbon dioxide fixation i. isolation and properties of an enzyme from pigeon liver catalyzing the reversible oxidative decarboxylation of l-malic acid. J. Biol. Chem..

[16] Bentle L., Lardy H.A. (1976). Interaction of anions and divalent metal ions with phosphoenolpyruvate carboxykinase. J. Biol. Chem..

[17] Schmittgen T.D., Livak K.J. (2008). Analyzing real-time PCR data by the comparative CT method. Nat. Protoc..

[18] Hill J.O., Wyatt H.R., Peters J.C. (2012). Energy balance and obesity. Circulation.

[19] Baker J.S., McCormick M.C., Robergs R.A. (2010). Interaction among skeletal muscle metabolic energy systems during intense exercise. J. Nutr. Metab..

[20] Westerblad H., Bruton J.D., Katz A. (2010). Skeletal muscle: Energy metabolism, fiber types, fatigue and adaptability. Exp. Cell Res..

[21] Horowitz J.F., Klein S. (2000). Lipid metabolism during endurance exercise. Am. J. Clin. Nutr..

[22] Kim J.-Y., Hickner R.C., Cortright R.L., Dohm G.L., Houmard J.A. (2000). Lipid oxidation is reduced in obese human skeletal muscle. Am. J. Physiol.-Endocrinol. Metab..

[23] Yki-Järvinen H. (2014). Non-alcoholic fatty liver disease as a cause and a consequence of metabolic syndrome. Lancet Diabetes Endocrinol..

[24] Van Herpen N., Schrauwen-Hinderling V. (2008). Lipid accumulation in non-adipose tissue and lipotoxicity. Physiol. Behav..

[25] Klaus S. (2004). Adipose tissue as a regulator of energy balance. Curr. Drug Targets.

[26] Lee M.R., Yang H.J., Park K.I., Ma J.Y. (2019). *Lycopus lucidus Turcz*. ex Benth. Attenuates free fatty acid-induced steatosis in HepG2 cells and non-alcoholic fatty liver disease in high-fat diet-induced obese mice. Phytomedicine.

[27] Fatani S., Itua I., Clark P., Wong C., Naderali E.K. (2011). The effects of diet-induced obesity on hepatocyte insulin signaling pathways and induction of non-alcoholic liver damage. Int. J. Gen. Med..

[28] Garg A., Misra A. (2002). Hepatic steatosis, insulin resistance, and adipose tissue disorders. J. Clin. Endocrinol. Metab..

[29] Zhou Y., Rui L. (2010). Major urinary protein regulation of chemical communication and nutrient metabolism. Vitam. Horm..

[30] Hui X., Zhu W., Wang Y., Lam K.S.L., Zhang J., Wu D., Kraegen E.W., Li Y., Xu A. (2009). Major urinary protein-1 increases energy expenditure and improves glucose intolerance through enhancing mitochondrial function in skeletal muscle of diabetic mice. J. Biol. Chem..

[31] Wu Y., Kim J.Y., Zhou S., Smas C.M. (2008). Differential screening identifies transcripts with depot-dependent expression in white adipose tissues. BMC Genom..

[32] Shin T.-Y., Kim S.-H., Suk K., Ha J.-H., Kim I., Lee M.-G., Jun C.-D., Lim J.-P., Eun J.-S. (2005). Anti-allergic effects of *Lycopus*
*lucidus* on mast cell-mediated allergy model. Toxicol. Appl. Pharmacol..

