# Supplementary material for: Lycopus lucidus Turcz Water Extract Ameliorates the Metabolic Disorder by Up-Regulated Major Urinary Protein Expression in High-Fat Diet-Induced Obesity
Source: Curr Issues Mol Biol. 2022 May 23;44(5):2417–30. doi: 10.3390/cimb44050165 (PMC9164051; doi:10.3390/cimb44050165)
Supplement: Supplementary file 1 [file cimb-44-00165-s001.zip › cimb-1733828-supplementary.pdf]

Supplementary Table S1. Experimental Diets

Ingredient (g)	ND	HFD	LT
Casein	200	265	265
Corn Starch	397.486	-	-
Sucrose	100	90	75
Dextrose	132	160	160
Cellulose	50	65.6	65.6
Soybean Oil	70	30	30
Lard	-	310	310
Mineral Mix[1]	35	48	48
Vitamin Mix[2]	10	21	21
Calcium phosphate, Dibasic	-	3.4	3.4
TBHQ, antioxidant	0.014	-	-
L-Cystine	3	4	4
cholin Bitartrate	2.5	3	3
Lycopus lucidus Turcz extract	-	-	15
Total (g)	1000	1000	1000

[1] AIN-93G- mineral mixture (Harlan Teklad Co., Madison, WI, USA).

[2]AIN-93G- vitamin mixture (Harlan Teklad Co., Madison, WI, USA).

Supplementary Table S2. Primer sequences used for RT-qPCR

Gene	Primer Direction	Primer Sequence
PPARa	Forward	
	Reverse	
CD36	Forward	
	Reverse	
Slc2a4	Forward	
	Reverse	
PGC-1a	Forward	
	Reverse	
Tfam	Forward	
	Reverse	
UCP2	Forward	
	Reverse	
SREBP1c	Forward	
	Reverse	
SREBP2	Forward	
	Reverse	
SCD1	Forward	
	Reverse	
FAS	Forward	
	Reverse	
Nrf1	Forward	
	Reverse	
Sirt1	Forward	5′-GGAGCAGATTAGTAAGCGGCTTG-3’
	Reverse	5′-GTTACTGCCACAGGAACTAGAGG-3’
Ppargc1α	Forward	5′-GAATCAAGCCACTACAGACACCG-3’
	Reverse	5′-CATCCCTCTTGAGCCTTTCGTG-3’
Lpl	Forward	5′-GACTCGCTCTCAGATGCCCTAC-3’
	Reverse	5′-GCCTGGTTGTGTTGCTTGCC-3’
Acc2	Forward	5′-GCTGCGGTCAAGTGTATGCG-3’
	Reverse	5′-CACTGATGCATTTGCCCTGG-3’
Cpt1	Forward	5′-ATCTGGATGGCTATGGTCAAGGTC-3’
	Reverse	5′-GTGCTGTCATGCGTTGGAAGTC-3’
Pdk4	Forward	5′-CCGCTTAGTGAACACTCCTTC-3’
	Reverse	5′-TCTACAAACTCTCGACAGGGCTTT-3’
Acat	Forward	5′-AGAAATCAAGCAAAGATCCA-3’
	Reverse	5′-AGGAGTCCTTGGGTAGTTGT-3’
Hmgcr	Forward	5′-TGTGGCCAGGAGTTTGGTGACTGA-3’
	Reverse	5′-TAAGATTCAACAACTCTGCTGACC-3’
Tnfα	Forward	5′-AAAGACACCATGAGCACAGAAAGC-3’
	Reverse	5′-GCCACAAGCAGGAATGAGAAGAG-3’
Tnfrsf12a	Forward	5′-GACCTCGACAAGTGCATGGACT-3’
	Reverse	5′-CGCCAAAACCAGGACCAGACTA-3’
Tnfrsf10b	Forward	5′-TGTGTCGATGCAAACCAGGCAC-3’
	Reverse	5′-GCCGTTTTGGAGACACACTTCC-3’
Gapdh	Forward	5′-AAGGTCATCCCAGAGCTGAA-3’
	Reverse	5′-CTGCTTCACCACCTTCTTGA-3’

Sirt1, sirtuin 1; Ppargc1α, peroxisome proliferative activated receptor, gamma, coactivator 1 alpha; Lpl, lipoprotein lipase; Acc2, acetyl-CoA carboxylase 2; Cpt1, carnitine palmitoyltransferase I; Pdk4, pyruvate dehydrogenase kinase 4; Acat, acetyl-CoA acetyltransferase; Hmgcr, 3-hydroxy-3-methylglutaryl-CoA reductase; Tnfα, tumor necrosis factor alpha; Tnfrsf12a, tumor necrosis factor receptor superfamily, member 12a; Tnfrsf10b, tumor necrosis factor receptor superfamily, member 10b; Gapdh, glyceraldehyde-3-phosphate dehydrogenase.
